# Authors' response: Comment on “clinicopathological features, survival outcomes, and appropriate surgical approaches for stage I acinar and papillary predominant lung adenocarcinoma”

**DOI:** 10.1002/cam4.4585

**Published:** 2022-02-09

**Authors:** Di Lu, Jianjun Yang, Xiguang Liu, Siyang Feng, Xiaoying Dong, Xiaoshun Shi, Jianxue Zhai, Shijie Mai, Jianjun Jiang, Zhizhi Wang, Hua Wu, Kaican Cai

**Affiliations:** ^1^ Department of Thoracic Surgery Nanfang Hospital, Southern Medical University Guangzhou China

Some concerns were raised related to our study in the comments, which was published earlier in Cancer Medicine “Clinicopathological features, survival outcomes, and appropriate surgical approaches for stage I acinar and papillary predominant lung adenocarcinoma” (Article ID: CAM43012, Article DOI: 10.1002/cam4.3012, Internal Article ID: 16708831). They raised that it was of little significance to investigate appropriate surgical procedures for patients with acinar predominant adenocarcinoma (ACN) and papillary predominant adenocarcinoma (PAP) because prediction derived from intraoperative frozen section (FS) for histologic patterns could not be precise enough, and the chemotherapy data in our study, which was from the Surveillance, Epidemiology, and End Results (SEER) database, might not be reliable enough.

For the first concern, the prognostic value of the International Association for the Study of Lung Cancer/American Thoracic Society/European Respiratory Society international multidisciplinary lung adenocarcinoma classification has been confirmed,^1^, ^2^, ^3^ thus, from the perspective of surgeons, it is necessary to investigate the appropriate surgical approaches of the subtype of lung adenocarcinoma to make a better scheme of surgical treatment and surveillance after operation. Although several studies have reported that prediction derived from FS for histologic patterns could not be precise enough,^4^, ^5^ it should be noted that the accuracy rate of FS for predicting ACN (76%) and PAP (85%) and interobserver agreement (κ = 0.481 and 0.527) was acceptable.^4^ And acinar pattern was the primary histological pattern that was most likely to be correctly identified on FS (sensitivity 87.6%; specificity 65.4%), while papillary predominant tumors also had moderate sensitivity (50%) and high specificity (96.6%).^5^ Besides, taking more sections during FS may help to improve the diagnostic accuracy.^4^ Furthermore, with the development of technology, some new techniques, such as inflation treatment for FS, may help to improve the diagnostic accuracy.^6^ Since our research indicated that segmentectomy was equivalent to lobectomy for stage I ACN while lobectomy remained the optimal procedure for stage I PAP, we believe that if these results could be confirmed by more data from other medical centers, there would be great demands for improving the accuracy of rapid intraoperative diagnosis. In addition, other methods, such as artificial intelligence and computed tomography, could also assist in diagnosis before operation.^7^, ^8^, ^9^ Last but not least, even though intraoperative FS did not give the correct result, our result could be referred to make and adjust the plan of treatment and surveillance after the operation, especially for those whose resected extension was insufficient.

As for the second concern, we agree that the chemotherapy data from the SEER database are incomplete. It is one of the limitations of our study, however, it is the best we can do, for which those patients who did not receive chemotherapy and those whose chemotherapy history was unknown could not be distinguished in SEER database. Besides, it should be noted that Qian et al reported that adjuvant chemotherapy was a prognostic factor only for patients with stage IB lung adenocarcinoma with solid or micropapillary predominant pattern, however, it did not contribute to both disease‐specific survival and recurrence neither in solid/micropapillary‐negative subgroup (disease‐specific survival: *p* = 0.421; recurrence: *p* = 0.189) nor in solid/micropapillary‐minor subgroup (disease‐specific survival: p = 0.098; recurrence: *p* = 0.886) after propensity‐score matching.^10^ In line with this, similar results were observed in other researches.^11^, ^12^ These data indicate that chemotherapy might have no effect on the survival of patient with stage I ACN or PAP. What is more, some studies with high quality also draw conclusions based on the data from SEER database, although some information, such as degree of differentiation and whether receive surgery or chemotherapy, was incomplete.^13^, ^14^ Furthermore, we are going to collect the data in our medical center to validate the conclusion in the future.

To sum up, it is necessary to investigate the proper surgical plan for patients with ACN or PAP for a better scheme of surgical treatment and surveillance after operation, although the accuracy of intraoperative FS for predicting the histologic patterns of lung adenocarcinoma is not satisfactory enough so far. Besides, we agree that chemotherapy data from the SEER database are incomplete, however, chemotherapy might have no effect on the survival of patient with stage I ACN and PAP.

Sincerely,

Kaican Cai.

Head of the Department of Thoracic Surgery,

Nanfang Hospital, Southern Medical University.

## CONFLICT OF INTEREST

The authors declare no competing financial interests.

## AUTHOR CONTRIBUTIONS

DL, JY, and XL wrote the manuscript. HW and KC revised the manuscript. All authors read and approved the final manuscript.

## Data Availability

The data that support the findings of this study are available from the corresponding author upon reasonable request.
